# Deep learning disconnectomes to accelerate and improve long-term predictions for post-stroke symptoms

**DOI:** 10.1093/braincomms/fcae338

**Published:** 2024-09-30

**Authors:** Anna Matsulevits, Pierrick Coupé, Huy-Dung Nguyen, Lia Talozzi, Chris Foulon, Parashkev Nachev, Maurizio Corbetta, Thomas Tourdias, Michel Thiebaut de Schotten

**Affiliations:** Groupe d'Imagerie Neurofonctionnelle, Institut des Maladies Neurodégénératives 5293, Centre National de la Recherche Scientifique (CNRS), University of Bordeaux, 33076 Bordeaux, France; Brain Connectivity and Behaviour Laboratory, Sorbonne Universities, 75006 Paris, France; University Bordeaux, Centre National de la Recherche Scientifique (CNRS), Bordeaux Institute Polytechnique de Bordeaux (INP), Laboratoire Bordelais de Recherche en Informatique (LaBRI), CNRS 5800, 33405 Talence, France; University Bordeaux, Centre National de la Recherche Scientifique (CNRS), Bordeaux Institute Polytechnique de Bordeaux (INP), Laboratoire Bordelais de Recherche en Informatique (LaBRI), CNRS 5800, 33405 Talence, France; Department of Neurology and Neurological Sciences, Stanford University School of Medicine, Stanford, CA 94305, USA; Institute of Neurology, University College London, WC1N 3AZ London, UK; Institute of Neurology, University College London, WC1N 3AZ London, UK; Clinica Neurologica, Department of Neuroscience, University of Padova, 32122 Padova, Italy; Padova Neuroscience Center (PNC), University of Padova, 32122 Padova, Italy; Venetian Institute of Molecular Medicine (VIMM), 32122 Padova, Italy; Centre Hospitalier Universitaire (CHU) de Bordeaux, Neuroimagerie Diagnostique et Thérapeutique, 33076 Bordeaux, France; University Bordeaux, National Institute of Health and Medical Research (INSERM), Neurocentre Magendie, U1215, 33076 Bordeaux, France; Groupe d'Imagerie Neurofonctionnelle, Institut des Maladies Neurodégénératives 5293, Centre National de la Recherche Scientifique (CNRS), University of Bordeaux, 33076 Bordeaux, France; Brain Connectivity and Behaviour Laboratory, Sorbonne Universities, 75006 Paris, France

**Keywords:** disconnectome, long-term predictions, stroke, deep-learning, white matter

## Abstract

This study investigates the efficacy of deep-learning models in expediting the generation of disconnectomes for individualized prediction of neuropsychological outcomes one year after stroke. Utilising a 3D U-Net network, we trained a model on a dataset of *N* = 1333 synthetic lesions and corresponding disconnectomes, subsequently applying it to *N* = 1333 real stroke lesions. The model-generated disconnection patterns were then projected into a two-dimensional ‘morphospace’ via uniform manifold approximation and projection for dimension reduction dimensionality reduction. We correlated the positioning within this morphospace with one-year neuropsychological scores across 86 metrics in a novel cohort of 119 stroke patients, employing multiple regression models and validating the findings in an out-of-sample group of 20 patients. Our results demonstrate that the 3D U-Net model captures the critical information of conventional disconnectomes with a notable increase in efficiency, generating deep-disconnectomes 720 times faster than current state-of-the-art software. The predictive accuracy of neuropsychological outcomes by deep-disconnectomes averaged 85.2% (*R*^2^ = 0.208), which significantly surpassed the conventional disconnectome approach (*P* = 0.009). These findings mark a substantial advancement in the production of disconnectome maps via deep learning, suggesting that this method could greatly enhance the prognostic assessment and clinical management of stroke survivors by incorporating disconnection patterns as a predictive tool.

## Teaser

Matsulevits *et al*. report substantial improvements in predicting stroke outcomes using a deep learning model. It efficiently and accurately generates individual lesion-specific white matter disconnectivity maps, referred to as ‘deep-disconnectomes’. These synthetic disconnectomes demonstrate superior predictive capabilities for long-term neuropsychological outcomes in stroke patients compared to the conventional ground truth.

## Graphical Abstract

**Graphical Abstract:**
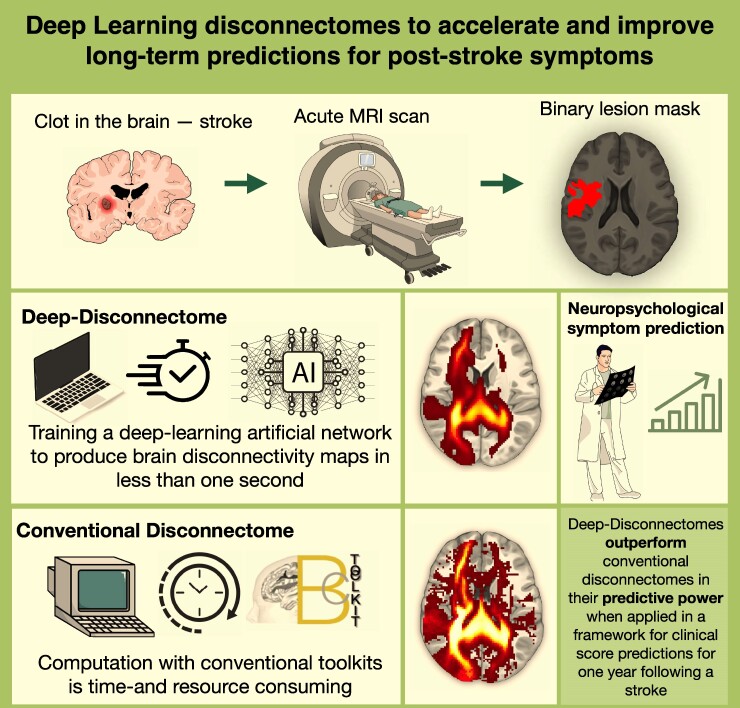

See Demeyere and J. Moore (https://doi.org/10.1093/braincomms/fcae364) for a scientific commentary on this article.

## Introduction

Over the last decades, a paradigm shift in neuroscience has emphasized the critical role of brain area interactions, with the functioning as a network of interconnected regions rather than segregated entities.^1^ As a result, white matter (WM) connections are recognized as fundamental building blocks of behaviour and cognition, and their disconnections can be estimated to facilitate personalized prediction. This is particularly relevant in the context of stroke that is going to damage a specific brain region but also disconnect several remote areas. Several studies have shown that incorporating information on the disconnection could help to anticipate the motor, anosognosic, or aphasic (language) outcome better compared to a volume- or location-based approach.^2-6^ This is even more relevant for post-stroke cognitive or neuropsychological impairment as these higher-order functions are likely dependent on the capacity of the brain to function as a network.^7^ Importantly, more than half of patients develop post-stroke cognitive impairment even after excellent functional recovery, which can have a significant impact on a patient's well-being and is strongly correlated with reduced societal reintegration and difficulty returning to work.^8,9^ Therefore, being able to anticipate the risk of developing such impairment following stroke could help to refer the patients to dedicated training to improve their outcome.

In this context, the search for new tools to capture the disconnection information following stroke represents a cutting-edge frontier. Whole brain connections—tractograms—can be reconstructed with diffusion MRI that estimates the direction of microscopic water molecules diffusion that preferentially follows the direction of the fibres.^10-13^ The acquisition of diffusion MRI is now widely feasible on modern MRI scanners and clinical settings. However, many parameters must be set to obtain diffusion scans suitable for reconstructing WM tracts. Moreover, the virtual dissection of WM tracts is often time-consuming and requires trained personnel. For these reasons, WM investigations are rarely integrated into clinical settings even during follow-up exams of stroke patients.

A first answer in providing more applicable WM reconstructions was to exploit tractograms of normative populations to estimate the probability of disconnections given a certain lesion in the brain. Accordingly, brain disconnection maps—disconnectomes—are derived from patients’ binary lesion masks, which could be easily segmented from clinical MRI scans conventionally acquired in hospitals.^14-16^ Such indirect brain disconnection patterns computed a few days after stroke showed first promising predictions of the long-term impairment on different neuropsychological tests.^17^ However, obtaining the disconnectome from any toolkit is still a resource-consuming process,^14-16^ which makes it less applicable in the prospect of possible clinical translation. Yet, it has never been tested whether deep learning could potentially make the complex and accurate 3D reconstruction of disconnected fibres a fast and automatic process while preserving clinical prediction accuracy. In this work, we apply and evaluate the performance of a trained deep-learning model for disconnectome reconstruction and stroke outcome prediction.

## Materials and methods

### Data for model training and testing

For the analysis, three different datasets were used. All patients have been previously reported by Talozzi *et al*.^17^ but were reused here to train and validate a new deep-learning-based disconnectome, later referred to as ‘deep-disconnectome’, and test its predictability. We trained the 3D U-Net^18^ model on 1333 synthetically created lesion-disconnectome dataset pairs (dataset 1). After training (80% of dataset 1) and validation (20% of dataset 1), we tested it on an out-of-sample dataset of 1333 natural stroke lesions (dataset 2). Additionally, we evaluated the predictive power of deep-disconnectomes to predict clinical scores following a recently published approach^17^ using stroke patients scanned 2 weeks after stroke and followed at 1 year with detailed clinical evaluations spanning from motor and language abilities to visuospatial, memory and sickness reports (dataset 3, later split in *N* = 119 for training and *N* = 20 for out-of-sample prediction). Details of the acquired scores can be found from Talozzi *et al*.^17^ For all these datasets, stroke lesions have been previously delineated manually and registered in the MNI space.^17,19^ More detailed information on the datasets used, as well as on the creation of the synthetic lesions used for the model training can be found in the Supplementary Table 1.

### General design of the analyses

The conventional disconnectomes were computed as previously reported by projecting each infarct mask on whole brain tractograms of 176 healthy participants whose 7T diffusion-weighted scans were obtained as part of the Human Connectome Project.^14,20,21^ Briefly, we used the BCBtoolkit which outputs a disconnectome as a map ranging from 0 to 1 according to the number of healthy participants who have a streamline passing through a voxel of the lesion mask.^14^ The ultimate goal of our new deep-learning approach is to be able to output such a diffusion/disconnection information but using only the lesion mask.

Furthermore, to capture the complexity of the 3D disconnectomes, we used a new formalism that we described recently^17,21^ and that consists of embedding the disconnection profiles within a 2D space called morphospace applying the uniform manifold approximation and projection (UMAP) for dimension reduction approach. The UMAP algorithm, based on the Riemannian geometry approximation and algebraic topology, constructs a high-dimensional representation of the input data and thereafter optimizes a low-dimensional graph while preserving the structural similarity of the data as much as possible.^22^ It is configured to find the shortest path between samples based on their similarity in the provided data structure.^22^ Hence, in the deep-disconnectome morphospace, patients with a similar disconnection profile cluster together while patients with different disconnections are located further apart. One advantage of the morphospace is that location within this space can be then correlated with any type of clinical outcome to identify territories at risk which we aim to compare between a morphospace built from conventional disconnectomes or the developed deep-disconnectomes.

### Deep learning 3D U-Net algorithm

**Figure 1:**
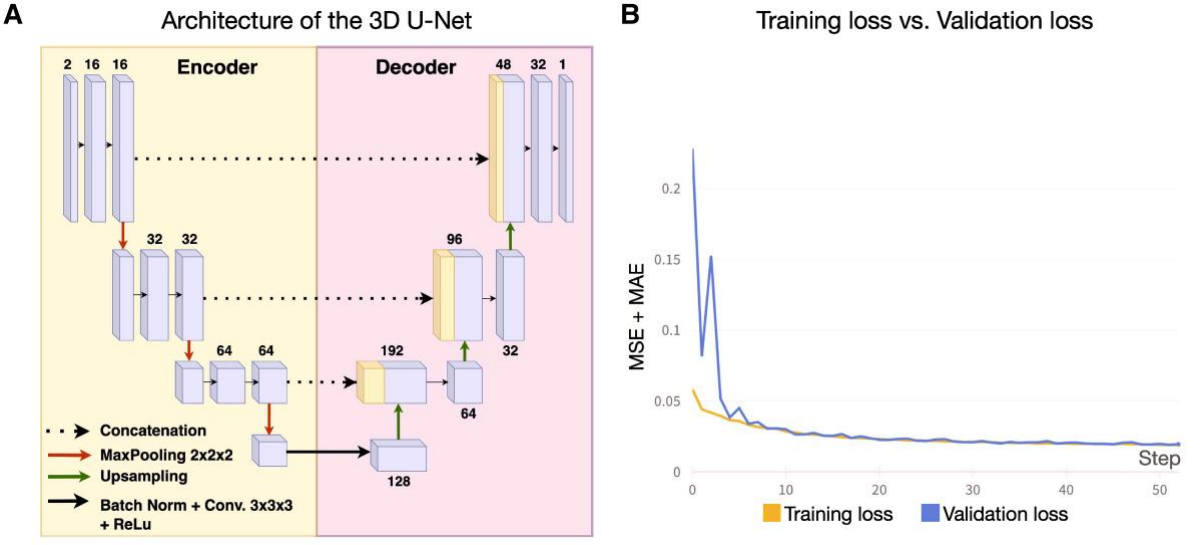
**Model architecture and training progress (A) Architecture of the 3D U-Net used for deep-disconnectome predictions.** The number above/below each block is the number of the used channels. (**B**) Training (80% of the dataset; *N* = 1066) and validation (20% of the dataset; *N* = 267) loss over the time course of the first 50 epochs was evaluated by adding the MSE and MAE.

We implemented a 3D U-Net network for predicting individual deep-disconnectomes from binary lesion masks (Fig. 1A). Our encoder architecture consisted of three convolutional blocks, whereas the decoder transposed convolutions in three upconvolutional blocks to reconstruct the original image resolution. The encoder modules’ convolutional blocks consisted of two 3D convolutional layers with batch normalisation and Leaky Rectified Linear Unit (ReLu) activation functions, followed by a max-pooling layer with a dropout rate of 0.5. In the encoding phase, the number of filters in each block was doubled with respect to the previous block being gradually increased from 16 to 64 in order to increase the capacity of the model to learn more complex features. At the same time, the spatial resolution was reduced by half with each max-pool operation. In the decoder module, the upconvolutional blocks combined the output of the previous block with the corresponding output from the contracting path. Each of the upconvolutional blocks consisted of an upsampling layer (using trilinear interpolation), a concatenation operation, a 3D convolutional layer with batch normalisation, and a ReLu activation function. In convolutional layers, the size of all convolutional kernels was 3 × 3 × 3. For all max-pooling layers, the pooling size was 2 × 2 × 2 with stride = 2. The spatial resolution of the output was doubled with each upsampling operation.

The model input consisted of two 3D channels of the volume size 96 × 96 × 96 (voxels at 2 mm^3^). The first channel contained the binary stroke lesion mask, and the second channel was the average disconnectome image computed from 1333 synthetic disconnectomes using *fslmaths* as part of the FSL package.^23^ The average disconnectome template was kept constant and was used to drive the model towards plausible disconnection outputs. During the data loading procedure, the images (91 × 109 × 91) underwent a size transformation containing interpolation to ensure equal dimensions of the images. In the evaluation, this transformation was reversed to compare the output of the model to the ground truth (GT). The model obtained its final output by a 3D convolutional layer, which was applied with a sigmoid activation function that mapped the network output to the intensity range of the conventional disconnectome images, the GT used for the training (i.e. between 0 and 1, representing the probability of a voxel to be disconnected). Voxels outside of an a priori calculated brain mask based on the 2 mm MNI152 T1^19^ were excluded from model training, evaluation and predictions. Local computational resources allowed for training with a batch size of four and a learning rate of 0.0003. During the training, the model updated its weights based on the sum of the MSE (mean squared error) and MAE (mean absolute error) loss functions. Combining these losses strikes a balance for penalising errors, allowing to capture fine details, as well as the overall structure in the predicted outcome.^24^ To minimize the loss, the model network weights were iteratively updated with a weight decay of 0.0001 using the Adam optimizer. The model training was programmed for a maximum of 300 epochs on an NVIDIA Quadro P4000 Graphics Processing Unit (GPU of 8GB), with an early stop of 50.

### Statistical analyses

#### Deep-disconnectomes and conventional disconnectomes comparison

To assess the level of similarities between deep-disconnectome and conventional disconnectomes, we first produced a pairwise Pearson *R*^2^ revealing the percentage of the variance of disconnection reproduced by the 3D U-Net.

To explore any systematic differences in terms of disconnected voxels, we contrasted frequency maps of disconnected voxels (from dataset 2), comparing the conventional disconnectomes and their corresponding deep-disconnectomes. To facilitate this comparison, it was necessary to binarize both sets of disconnectomes. This binarisation was done by applying a threshold of 0.0001 to address the non-zero voxel values produced by the deep-disconnectome model, ensuring that our comparison was based on equivalent binary masks. This threshold preserved the GT data integrity and allowed for a direct and fair comparison of the embedding properties between the two disconnectome types. Subsequently, the embedding properties were compared across the two disconnectome methods. To compare the coordinates in the disconnectome morphospace, we computed the average Euclidean distances for 139 coordinate pairs (corresponding to dataset 3) across deep-disconnectomes and conventional disconnectomes. This comparison allowed us to explore the relative distances between data points in the embedding space.

#### Prediction of neuropsychological scores one year after stroke

Achieving clinical prediction is the ultimate goal of our investigation of stroke disconnections. To test and compare the potential in clinical score prediction, we compared the deep-disconnectomes and the conventional disconnectomes performances in the following domains: motor, language, visuospatial attention, visuospatial memory, verbal memory, pain and sickness (detailed lists of the 86 scores can be found in the Supplementary Table 2). While *R*^2^ serves as our primary outcome measure reflecting how well the prediction model of neuropsychological scores captures the variability in the data, we also included MAE normalized by the maximum score obtained in the neuropsychological evaluation (MAE %) as a measure of accuracy (1 − MAE %). The MAE % offers a complementary perspective on model performance by quantifying the average magnitude of prediction errors relative to the scale of the observed data. While offering insights into the accuracy of individual predictions and their closeness to the true values, the MAE % may show a disparity with the *R*^2^ score, which primarily focuses on capturing variability within the data. By reporting both *R*^2^ and MAE %, we offer a more comprehensive evaluation of the disconnectomes’ performance within our predictive framework.


$$\mathrm{MAE}\text{\%}=\;\frac{\sum_{i=1}^{N}\;|\mathrm{measured}-\mathrm{predicted}|/N}{\max(\mathrm{score})}$$

For this purpose, we used dataset 3, recruited at the School of Medicine at Washington University in St. Louis. To test the predictive quality of the deep-disconnectomes obtained from the 3D U-Net model, we replicated the analysis and validation procedures of the work of Talozzi *et al*.^17^ First, 1333 disconnectomes of dataset 2 were used to compute the 2D UMAP^22^ morphospace based on the standard computation of disconnectomes through the BCBToolkit software.^14^ Following this step, disconnectome maps from the training subset of dataset 3 (*n* = 119) were imported into the UMAP morphospace, and their localisation was statistically correlated to the individual neuropsychological scores on the *N* = 86 different behavioural and cognitive scores (see Supplementary Table 2). Converting the UMAP coordinates of the patients into 2D nifty images allowed us to run pixel-wise Pearson correlations between the patient probability of localisation and the neuropsychological scores of these patients. Only values surpassing the threshold for medium effect size *R* > 0.2 were considered informative. To handle multiple clusters of voxels that survived this threshold, a principal component analysis (PCA) was run to capture the greatest source of patient coordinate variability. Based on the first three PCA components, a multiple regression model (Python *scikit-learn* package) was evaluated to predict each neuropsychological score. Note that the datasets used in the present paper are the same as by Talozzi *et al*.,^17^ the only difference being the method of how the disconnectomes were obtained: in previous work^17^ using BCBToolkit software, here using a trained 3D U-Net model. Hence, conventional disconnectomes and deep-disconnectomes are systematically comparable. We finally compared their predictive power by running a paired *t*-test (2-tails) on the obtained *R*^2^ scores for each neuropsychological score prediction.

## Results

### Accuracy in deep-disconnectome reconstruction

Training the model for 260 epochs took 33.527 h on the NVIDIA Quadro P4000 GPU. The resulting training loss and validation loss were 0.01652 and 0.01767, respectively (Fig. 1B). After training the model, the average time required to generate a deep-disconnectome from a binary lesion mask was 0.642 s, which is significantly faster compared to the conventional disconnectome generation time of 480 s/8 min (standard computer setup with an Intel Core-i7 processor).

**Figure 2:**
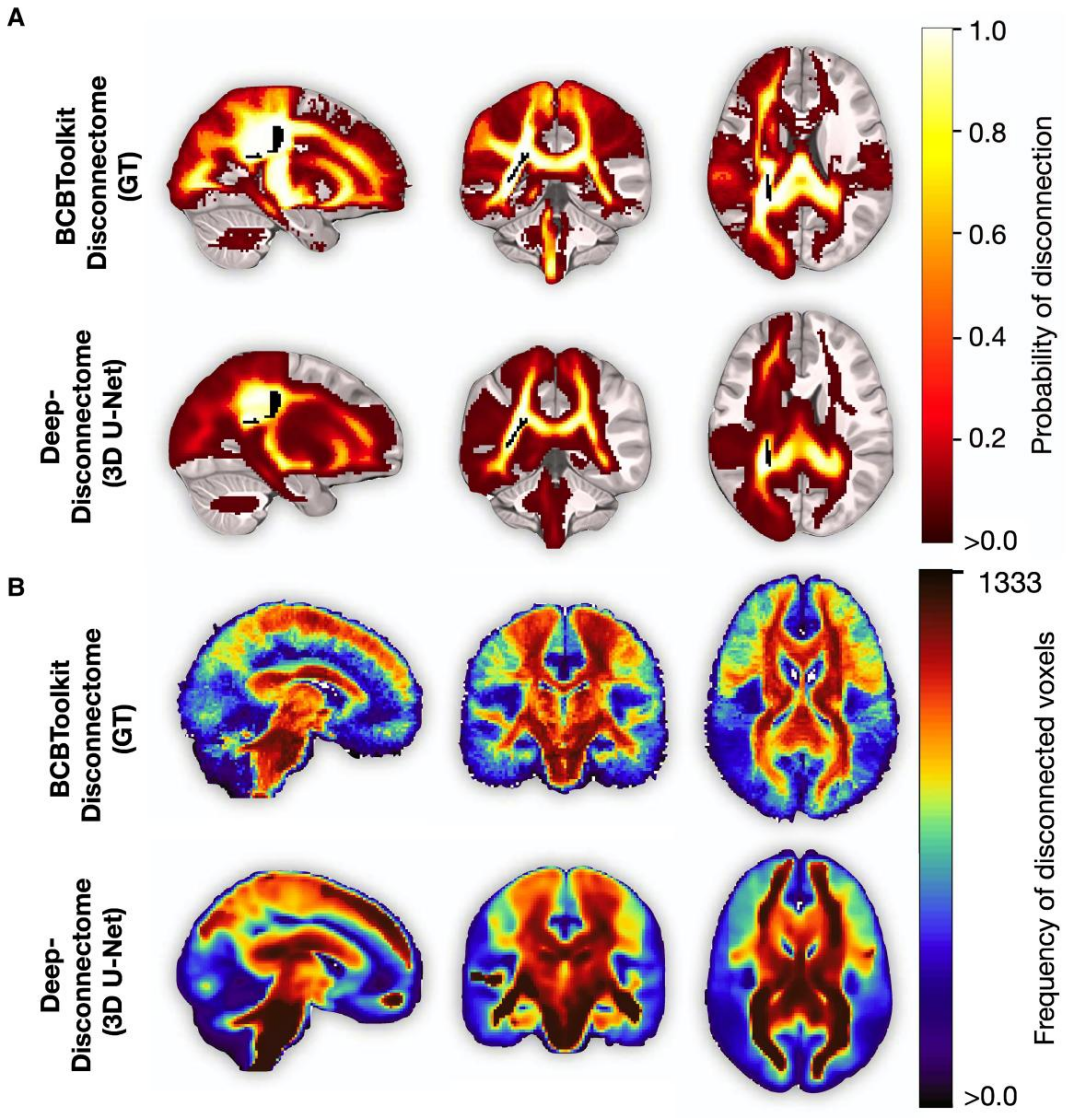
**Deep-disconnectomes accurately resemble the conventional disconnectomes.** (**A**) Visual comparison of a representative (*N* = 1) out-of-sample patient conventional disconnectome obtained from the BCBToolkit software and used as the GT in the model training (*top*) with its 3D U-Net predicted deep-disconnectome (*bottom*). (**B**) Frequency maps of the disconnected voxels obtained by comparing binarized conventional (*N* = 1333) and binarized deep-disconnectomes (*N* = 1333) (dataset 2). Higher values indicate the voxels to be disconnected more frequently, while lower values indicate less frequent disconnections of these particular voxels.

Figure 2A shows an out-of-sample individual example of the conventional and deep-learning version of the disconnectome for the same subject, together with this patient’s stroke lesion. The overall goodness of fit metric, *R*^2^, reached 0.82, meaning that the 3D U-Net model is able to explain, on average, more than 80% of the variance in the predicted image based on the input data. Visual comparison of the predicted and the GT disconnectomes demonstrates the reliability of the predictions to capture the relevant features and resemble the conventional disconnectome (Fig. 2A). Comparing the frequency maps of disconnected voxels between conventional disconnectomes and the corresponding deep-disconnectomes further reveals the disconnected structures that are reliably captured alongside the less well-captured structures. The frequency map visualisation (Fig. 2B) demonstrates significantly higher values for the core long WM structures and lower values, hence an underestimation of short, U-shaped association fibres located near the surface.

### Deep-disconnectome predictivity power for neuropsychological scores

**Figure 3:**
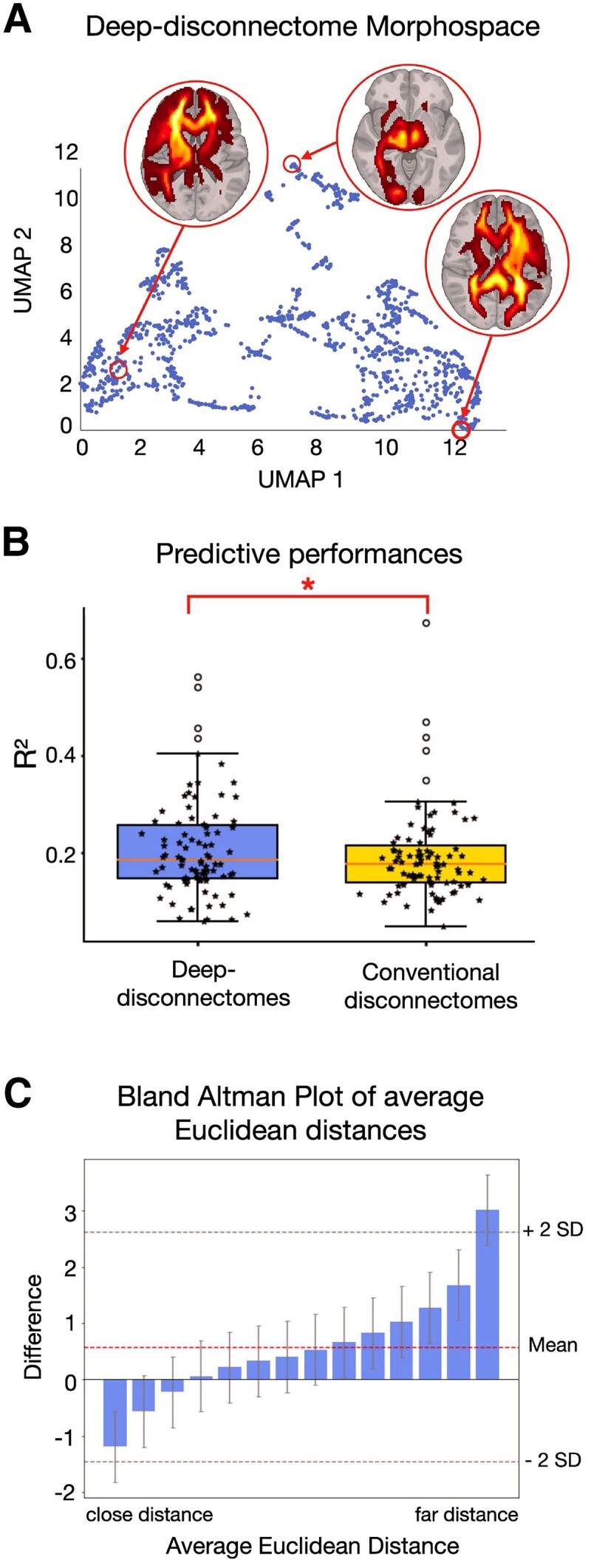
**Increased predictive performance with the deep-disconnectome.** (**A**) UMAP morphospace embedding of *N* = 1333 deep-disconnectomes with three exemplary deep-disconnectomes in different locations of the latent space. (**B**) The boxplot shows all the *R*^2^ for the predictions for stroke survivors (dataset 3, *N* = 119) across *N* = 86 neuropsychological scores for the framework using deep-disconnectomes compared to the framework using disconnectomes obtained by BCBToolkit. The boxes represent the quartiles, the whiskers indicate the distribution, and the outliers are marked as dots. Inside the boxes, the median is visualized by the solid line. The *P*-value obtained from a paired *t*-test (2-tails): *P < 0.01 shows a significant difference (*t*(85) = −1.663, *P* = 0.009). (**C**) The Bland Altman plot shows the difference in the average Euclidean distances between the conventional disconnectome and the deep-disconnectome for each of the *N* = 139 disconnectomes (dataset 3) to the rest of the data points in their given morphospace. Deep-disconnectomes demonstrate a better dissociation within the clusters (for close distances), as indicated by the negative differences. For far distances of the data points in the morphospace, deep-disconnectomes are located closer to each other than conventional disconnectomes, as indicated by the positive difference value. The error bars represent 95%-confidence intervals. The middle dotted line represents the mean, and the upper and lower dotted lines represent 2 standard deviations (SD) in each difference direction (positive and negative).

We imported the deep-disconnectomes derived from the 1333 stroke lesions of dataset 2 into the UMAP embedding,^22^ obtaining a novel morphospace for deep-disconnectomes (Fig. 3A).

To test the deep-disconnectome predictions of neuropsychological scores and compare these to the predictions of the conventional disconnectomes, we embedded *N* = 119 patients of dataset 3 into the novel morphospace and followed the analysis steps described by Talozzi *et al*.^17^ First, we converted each patient’s coordinates in the morphospace into the according probabilities of localisation. Subsequently, we calculated Pearson correlations between the patient’s locations in the morphospace and associated it with the individual’s neuropsychological performance. Based on the effect size associations (all |*R*| > 0.2), we extracted territories in the morphospace for each neuropsychological score that were linked to a certain disconnectivity profile. From the three most informative territorial clusters, performing a PCA, we obtained weights indicating the patient’s probability of localisation within each cluster and used these as inputs for a multiple regression analysis. With this method, we were able to predict *N* = 86 different behavioural and cognitive scores 1 year post-stroke for all the newly embedded patients. An average prediction accuracy of 85.7% and an average *R*^2^ = 0.208 were achieved.

For comparison, the average accuracy and *R*^2^ for the exact same predictions with a morphospace obtained using disconnectomes computed by the BCBToolkit software reached 83.7% and *R*^2^ = 0.191, respectively. A statistical pairwise comparison of the *R*^2^ scores resulted in (*t*(85) = −1.663, *P* = 0.009). Hence, computing the disconnectomes using the deep-learning model and obtaining a corresponding morphospace for predicting neuropsychological scores significantly increases the accuracy of the resulting scores by 1.5% on average (Fig. 3B). Interestingly, in both cases, when using conventional disconnectomes as well as when using deep-disconnectomes, the best domain to predict was the motor domain (average *R*^2^_Deep-Disconnectome Motor_ = 0.31, average *R*^2^_Conventional Disconnectome Motor_ = 0.28), whereas the least accurate domain to predict was visuospatial memory (VM) (average *R*^2^_Deep-Disconnectome VM_ = 0.11, average *R*^2^_Conventional Disconnectome VM_ = 0.16). Overall, the utilisation of the deep-disconnectome improved the *R*^2^ accuracy of 54 out of the 86 test scores.

To perform an out-of-sample validation, *N* = 20 stroke survivors from dataset 3 who were not included within the prediction training phase, were used as independent model validation. We outputted the deep-disconnectomes from the segmented binary stroke lesion and predicted their corresponding neuropsychological scores. The out-of-sample predictions achieved an average accuracy of 80.67%, with an *R*^2^ = 0.143. In comparison, the previous attempt with conventional disconnectomes^17^ reached an average accuracy of 80.35% with a lower average *R*^2^ = 0.121 (Fig. 1).

To understand why the score prediction of the deep-disconnectome outperforms the conventional disconnectome, a systematic comparison across the embedding coordinates was performed for each patient. We assessed the structure of the latent space by means of the average Euclidean distance between each embedding set of coordinates (UMAP 1 and 2) against the rest of the embedding points. This calculation reveals a higher distance within clusters of the deep-disconnectome when compared to the conventional disconnectome (see Supplementary Fig. 2 for a side-by-side comparison of the two spaces). Thus, this suggests a greater differentiation for deep-disconnectomes derived from similar stroke lesions. Reversely, the distance between clusters is smaller for the deep-disconnectome than in the conventional disconnectome framework, suggesting a more uniform spread within the morphospace. These differences may have improved the segregation between similar profiles of WM damage (i.e. in the same cluster) and, accordingly, could have led to better modelling of fine differences within the same neuropsychological assessment (Fig. 3C).

## Discussion

The deep learning 3D U-Net model we trained can generate accurate maps of brain disconnectivity—deep-disconnectomes—derived from a simple binary lesion mask. These generated disconnectivity maps have a clinical predictive power towards long-term impairment following a stroke that is even slightly higher than conventionally computed disconnectome maps. The validation of the deep-disconnectomes’ capacity to accurately predict neuropsychological scores demonstrates its potential to be released as an AI-driven tool for clinical applications.

Although our deep learning model generates disconnectome-like images, it does not capture exactly the same patterns as the conventionally computed disconnectomes. It can be speculated that the 3D U-Net model is potentially getting rid of noise while producing deep-disconnectomes, allowing for more concise information to be forwarded into the UMAP framework, consequently yielding more accurate predictions. Our investigation confirmed this speculation, revealing that deep-disconnectomes emphasize the core WM structures while exhibiting comparatively lower capacity in capturing short, more detailed, U-shaped fibres. Furthermore, we compared the average Euclidean distances of deep-disconnectomes and conventional disconnectomes to all the other points in their respective morphospace. We detected that deep-disconnectomes are better segregated within clusters of near data points. This means that deep-disconnectomes with small Euclidean distances (showing similar disconnectivity patterns) are dissociated better than conventional (BCBtookit) disconnectomes. On the contrary, deep-disconnectomes with large Euclidean distances (showing completely different disconnectivity patterns) are less different than conventional disconnectomes (which might not impact the predictive performances as these disconnectivity patterns are already well segregated).

Time and required computational capacity for obtaining an output is a common advantage of deep-learning methods, which are time-demanding in the training phase but have the tremendous gain of being fast when applied to new instances: a perfect fit for precision medicine. Using our trained deep-learning model takes <10 MB of free space on the operator’s device, and a deep-disconnectome can be outputted within <1 s. Usually, programmes that are able to compute brain disconnectomes^14-16^ require at least 1,5 GB up to 10 GB of free memory space, taking ∼720 times longer to create an output (0.642 s for the deep-disconnectome in comparison to 480 s for the conventional disconnectome).

While producing deep-disconnectomes with a trained AI model makes obtaining the otherwise processing-heavy data faster, the potential of its application goes beyond the advantage of gaining time. They outperform the conventional disconnectomes when applied in a framework for neuropsychological score predictions. Seemingly, the UMAP algorithm extracts features from the deep-disconnectomes in a more precise manner, which leads to better regression coefficients and, consequently, to better predictions for clinical test outcomes. Nonetheless, it is important to consider insights from the utilisation of direct measures of connectivity disruption, which suggest that individual-specific (dis-)connectomes may provide even finer-grained predictions of neuropsychological outcomes, albeit at the expense of the practicality and general applicability of such measures.^25^ How the slight improvement in predictive performances observed herein could impact clinical care will have to be tested in the future. However, our approach could be used as an awareness system to rapidly inform the caregivers of patients at risk for developing specific long-term impairments.

Finally, this framework makes obtaining disconnectivity data more accessible and, by that, consequently speeds up scientific progress. However, while integrating deep learning into various domains holds immense potential, its application still faces constraints. One prevailing limitation, which also pertains to our study, is the issue of generalizability. The efficacy of an algorithm heavily relies on the quality and representativeness of the training data it receives.^26^ Consequently, ensuring a diverse and comprehensive dataset becomes crucial for maximising the model's ability to generalize effectively. Within the realm of medical diagnosis, this challenge is particularly pronounced, exacerbated by biases and stringent privacy policies that restrict data availability as well as typical bias in the distribution of clinical data.^27^ However, in the specific context of disconnectomes, we are fortunate to have addressed this limitation by training our model on a broad sample of diverse synthetically generated lesions. Leveraging the capabilities of the BCBtoolkit, we can generate an abundance of training data by computing disconnectomes. Here, we generated 1333 such synthetic data as a symmetric number of our 1333 real patient’s data but future refinement could use any desired amount of data. This unique advantage augments our framework and unlocks novel opportunities for further exploration. The deep-disconnectome is theoretically able to capture the pattern of disconnection for any focal lesion (such as focal multiple sclerosis lesions, brain tumours and post-traumatic lesions). In future work, we can extend the model's training to include alternative focal lesions and examine the correlation between deep-disconnectome outcomes and associated neuropsychological manifestations. Incorporating actual patient data with alternative focal lesions into our deep-learning model would enhance its overall generalizability and establish a more comprehensive foundation for predicting neuropsychological scores following brain damage.

Overall, the achievement of accurately predicting deep-disconnectomes from binary lesion masks represents a significant initial milestone. This not only enriches the realm of neuroscience but also fosters the convergence of computer science with other disciplines, thereby pioneering advancements.

## Supplementary Material

fcae338_Supplementary_Data

## Data Availability

The trained model, the codes used, and exemplary files can be found at https://github.com/annamatsulevits/deep-disconnectome.
