# Supplementary material for: Deep learning disconnectomes to accelerate and improve long-term predictions for post-stroke symptoms
Source: Brain Commun. 2024 Sep 30;6(5):fcae338. doi: 10.1093/braincomms/fcae338 (PMC11503950; doi:10.1093/braincomms/fcae338)
Supplement: fcae338_Supplementary_Data [file fcae338_supplementary_data.docx]

**Supplementary Material**

1. *Datasets for model training and testing*

Our deep-learning framework was trained on a dataset of N=1333 synthetic lesions and their corresponding disconnectomes (Dataset 1) and subsequently tested for real stroke lesions (Dataset 2). The synthetic stroke lesions were initially computed by Thiebaut de Schotten, Foulon, and Nachev (1) to assess the spatial distribution of stroke impact on cognition. For that, they produced more than one million synthetic stroke lesion masks to sample out 1333 lesions matching in hemispheric lateralization and voxel size with natural lesions but randomly distributed in the brain. The probability of disconnectivity for each voxel in the brain was calculated with the BCBToolkit (2), and the obtained disconnectomes were used as ground truth to train our model. Using synthetic lesions had two main benefits: Firstly, we circumvented the eventual problem of insufficient training data since we could produce any desired number of synthetic lesion-disconnectome pairs. Secondly, the diversity of the training data increased, theoretically also increasing the generalizability of the trained model. During the training of our 3D U-Net, we used a split 80%/20% of synthetic data (Dataset 1) for, respectively, training and validation.

Subsequently, we used the N=1333 lesion masks segmented from patients recruited by the University College London Hospitals (Dataset 2) to test our trained deep-learning model. This patient cohort was recruited as part of a study approved by the West London and GTAC Research Ethics Committee. MRI scans (1.5 T and 3 T) of patients with acute ischemic stroke were acquired across several scanners between 1-2 weeks following the stroke (3). Patients (56% male) were, on average, 64 ± 16 years old (age range: 18–97 years).

Finally, we utilized the deep-disconnectomes of the stroke lesion masks of Dataset 3 for training the UMAP algorithm and correlating the localization of the patients with neuropsychological scores (Dataset 3.1, N=119), as well as the out-of-sample testing (Dataset 3.2, N=20). The dataset was collected at the School of Medicine of the Washington University, St. Louis (USA). MRI scans of patients with acute ischemic stroke were acquired within 14 ± 8 days (Dataset 3.1) and 13 ± 4 days (Dataset 3.2) after stroke. In Dataset 3.1, patients (54.6% male) were, on average, 54 ± 11 years old (age range: 19–83 years), in Dataset 3.2., patients (60% male) were, on average 58 ± 12 years old (age range: 34–95 years). All datasets used in this work are private datasets. A waiver from the owners was obtained prior to the study. For more details on the used datasets, see Table 1.

**Supplementary Table 1:** Information on the used datasets

|  | Dataset 1 | Dataset 2 | Dataset 3.1 | Dataset 3.2 |
| --- | --- | --- | --- | --- |
| Recruitment site | Synthetically produced (Thiebaut de Schotten, Foulon, and Nachev (2020)) | University College London Hospitals, London (UK) | School of Medicine of the Washington University, St. Louis (USA) | School of Medicine of the Washington University, St. Louis (USA) |
| Conducted analyses | 3D U-Net training | 3D U-Net testing, deep-disconnectome production, and UMAP space creation | UMAP training, 86 neuropsychological scores | out-of-sample testing of predictions, 86 neuropsychological scores |
| **Demographics** |  |  |  |  |
| N | 1333 synthetic lesions | 1333 real lesions | 119 | 20 |
| Males/females, *n* | NA | 748/585 | 65/54 | 12/8 |
| Age, years | NA | 64 ± 16 (18–97) | 54 ± 11 (19–83) | 58 ± 12 (34–95) |
| Education, years | NA | NA | 13.2 ± 2.5 (5–20) | 13.7 ± 2.6 (9–19) |
| Right-handed/left-handed, *n* | NA | NA | 109/10 | 17/3 |
| **MRI** |  |  |  |  |
| Chronology | NA | 1–2 weeks after stroke | 14 ± 8 days after stroke | 13 ± 4 days after stroke |
| Dominant lesion site | 44% right hemisphere  56% left hemisphere | 44% right hemisphere  56% left hemisphere | 46% right hemisphere  54% left hemisphere | 40% right hemisphere  60% left hemisphere |
| **Neuropsychological assessment** |  |  |  |  |
| Delay after stroke onset, days | NA | NA | 393 ± 56 | 385 ± 22 |
| Delay after MRI scan, days | NA | NA | 379 ± 57 | 1. ± 22 |

1. *Neuropsychological scores and domains*

**Supplementary Table 2**: Neuropsychological score abbreviations. Among the adopted examination tests: ARAT=Action Reaction Arm total test, AROM= Active Range Of Motion, SVFT= Standard Verbal Fluency Test, BIT= Behavioural Inattention Test, BDAE=Boston Diagnostic Aphasia Examination, Mes-USCT=Mesulam Unstructured Symbol Cancellation Test, BVMT=Brief Visuospatial Memory Test-revised, HVLT= the Hopkins Verbal Learning Test-revised, SA-SIP=Stroke-Adapted Sickness Impact Profile.

CD = Conventional Disconnectome

DD = Deep Disconnectome

***** Table adapted from Talozzi et al (2023)

| **Domain** | **Neuropsychological score (test battery)** | **R^2^ – CD** | **R^2^ – DD** | **Acc (%) - CD** | **Acc (%) - DD** |
| --- | --- | --- | --- | --- | --- |
| Motor | Left hand grasp (ARAT)  Left hand grip (ARAT)  Left hand pinch (ARAT)  Left hand grip strength  Left hand peg replacement  Left shoulder flexion (AROM)  Left wrist extension (AROM)  Right hand grasp (ARAT)  Right hand grip (ARAT)  Right hand pinch (ARAT)  Right hand grip strength  Right hand peg replacement  Right shoulder flexion (AROM)  Right wrist extension (AROM)  Walking combined score | 0.44  0.28  0.47  0.25  0.41  0.35  0.19  0.27  0.31  0.28  0.14  0.20  0.28  0.15  0.12 | 0.46  0.32  0.54  0.28  0.44  0.34  0.16  0.38  0.40  0.34  0.17  0.24  0.31  0.21  0.09 | 84.36  86.27  79.09  80.28  83.11  86.21  83.02  88.77  88.97  85.76  82.64  83.99  89.48  86.00  85.22 | 90.60  90.79  88.51  81.16  85.21  87.74  83.34  91.65  92.03  89.70  82.88  84.23  89.59  86.60  86.16 |
| Language | Sentence reading (BDAE)  Animal name fluency (SVFT)  Picture naming (BDAE)  Performing listen commands (BDAE)  Nonword repetition (BDAE)  Comprehension of read sentence (BDAE)  Comprehension of listen word (BDAE) | 0.17  0.17  0.12  0.27  0.22  0.10  0.16 | 0.15  0.16  0.16  0.28  0.24  0.11  0.06 | 91.71  86.96  88.36  92.17  82.67  88.90  97.06 | 90.83  86.10  90.42  92.23  84.11  88.77  96.41 |
| Visuospatial attention | Center of cancellation (BIT)  Left misses (BIT)  Right misses (BIT)  Total misses (BIT)  Center of cancellation (Mes-USCT)  Left misses (Mes-USCT)  Right misses (Mes-USCT)  Total misses (Mes-USCT)  Accuracy average (Posner)  Accuracy disengagement (Posner)  Accuracy left invalid (Posner)  Accuracy left valid (Posner)  Accuracy right invalid (Posner)  Accuracy right valid (Posner)  Accuracy validity (Posner)  Accuracy visual effect (Posner)  Reaction time average (Posner)  Reaction time disengagement (Posner)  Reaction time left invalid (Posner)  Reaction time left valid (Posner)  Reaction time right invalid (Posner)  Reaction time right valid (Posner)  Reaction time validity (Posner)  Reaction time visual effect (Posner)  Subbing average (Posner)  Subbing disengagement (Posner)  Subbing left invalid (Posner)  Subbing left valid (Posner)  Subbing right invalid (Posner)  Subbing right valid (Posner)  Subbing validity (Posner)  Subbing visual effect (Posner) Figure bias (BVMT) | 0.16  0.23  0.24  0.26  0.19  0.18  0.13  0.18  0.19  0.22  0.10  0.30  0.19  0.24  0.13  0.14  0.13  0.19  0.13  0.15  0.14  0.23  0.18  0.20  0.17  0.18  0.10  0.18  0.15  0.15  0.18  0.20 | 0.24  0.27  0.14  0.21  0.20  0.14  0.14  0.13  0.26  0.34  0.26  0.28  0.32  0.32  0.22  0.25  0.18  0.25  0.18  0.16  0.17  0.19  0.23  0.27  0.17  0.20  0.21  0.19  0.16  0.18  0.22  0.22 | 89.59  84.20  87.69  86.00  86.87  91.02  90.80  91.64  97.16  66.37  95.82  96.48  95.65  97.06  53.19  80.57  89.29  79.38  88.39  87.72  89.18  88.73  75.88  73.85  89.73  68.67  89.11  89.12  89.00  89.06  80.56  80.49 | 88.73  87.13  88.11  86.28  88.77  91.40  91.80  92.38  96.56  76.77  94.99  94.66  95.92  96.73  60.58  93.90  91.60  87.29  89.95  91.08  92.01  89.59  86.40  90.36  90.44  66.78  91.76  91.71  90.42  91.37  80.82  92.63 |
| Visuospatial memory | Figure bias (BVMT)  Figure delayed recall (BVMT)  Figure delayed recall t-score (BVMT)  Figure recognition discrimination (BVMT)  Figure false alarm (BVMT)  Figure recognition hit (BVMT)  Figure immediate recall (BVMT)  Figure immediate recall t-score (BVMT)  Figure learning (BVMT)  Figure percent retained (BVMT) | 0.25  0.11  0.20  0.11  0.12  0.19  0.14  0.15  0.08  0.21 | 0.12  0.07  0.11  0.11  0.06  0.16  0.09  0.08  0.14  0.14 | 86.76  74.56  73.39  85.78  88.00  93.10  76.64  76.51  76.11  91.98 | 90.61  78.55  78.80  87.51  82.75  93.22  79.96  78.69  79.64  93.10 |
| Verbal memory | Word recall (HVLT)  Word recall t-score (HVLT)  Word recognition (HVLT)  Word recognition t-score (HVLT)  Word recognition related false alarms (HVLT)  Word recognition unrelated false alarms (HVLT)  Word recognition false positive (HVLT)  Word recognition hits (HVLT)  Word immediate recall t-score (HVLT)  Word learning (HVLT)  Word recall retained (HVLT) | 0.12  0.10  0.16  0.16  0.13  0.67  0.17  0.09  0.05  0.10  0.18 | 0.06  0.08  0.16  0.14  0.16  0.56  0.19  0.17  0.06  0.17  0.18 | 74.90  72.90  85.47  77.05  80.67  93.27  80.62  91.30  82.66  74.11  83.32 | 78.58  75.38  87.07  80.02  82.36  94.95  83.11  91.82  84.75  77.27  85.28 |
| Pain | Pain | 0.29 | 0.29 | 82.35 | 87.28 |
| Sickness | Alertness behaviour (SA-SIP)  Ambulation (SA-SIP)  Body care and movement (SA-SIP)  Communication (SA-SIP)  Emotional behavior (SA-SIP)  Household (SA-SIP)  Mobility (SA-SIP)  Physical function (SA-SIP)  Psychosocial (SA-SIP)  Social (SA-SIP) | 0.10  0.16  0.19  0.15  0.21  0.11  0.19  0.15  0.20  0.15 | 0.09  0.19  0.16  0.15  0.24  0.15  0.26  0.13  0.23  0.15 | 69.44  71.98  75.84  71.99  78.34  72.08  75.65  80.17  79.90  77.91 | 67.82  70.07  75.47  73.57  78.76  71.07  75.74  79.97  78.99  77.45 |


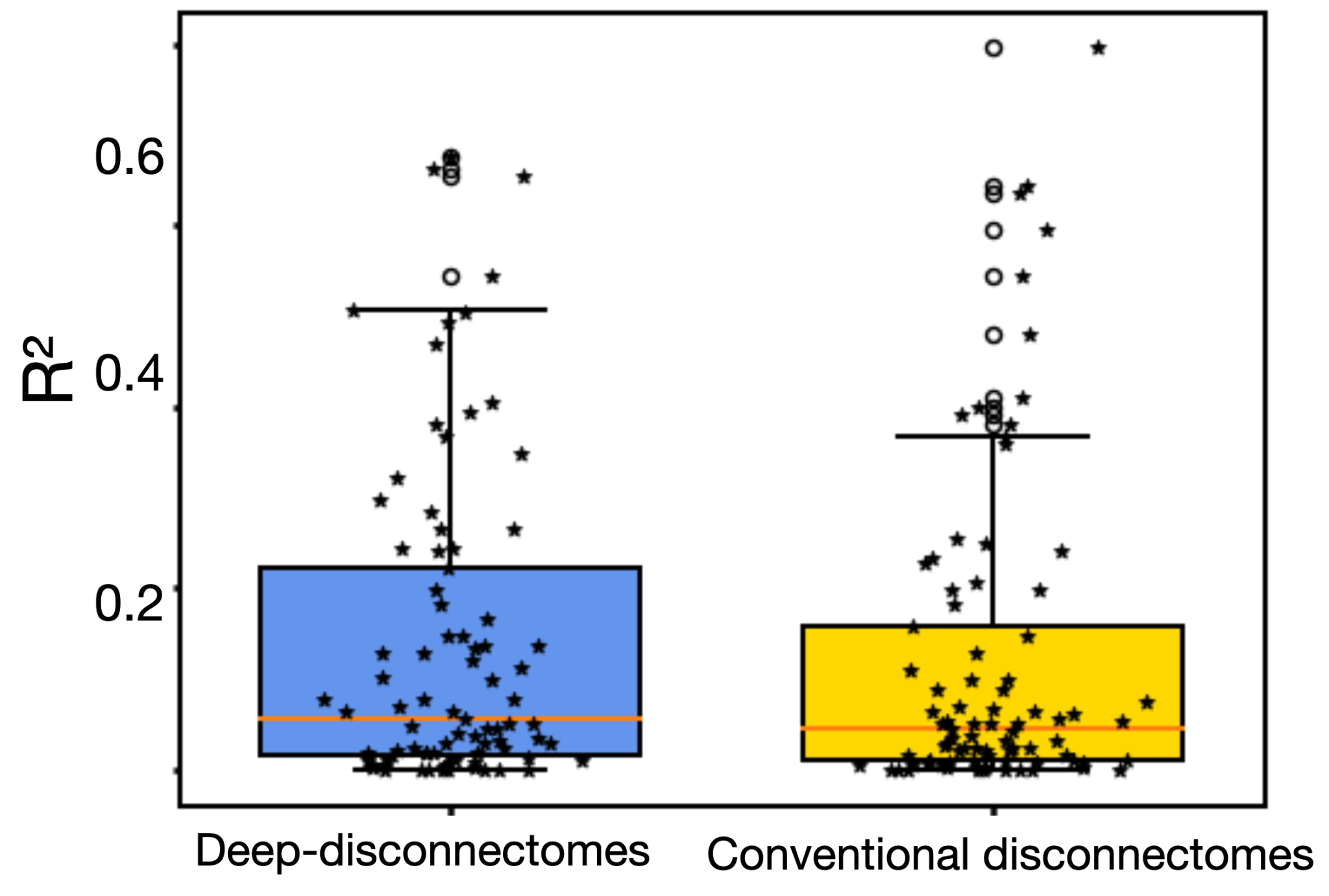


**Supplementary Figure 1:** The boxplot shows all the R² for the predictions of the validation set (N=20) across N=86 neuropsychological scores for the framework using deep-disconnectomes compared to the framework using disconnectomes obtained by BCBToolkit. The boxes represent the quartiles, the whiskers indicate the distribution, and the outliers are marked as white dots. Inside the boxes, the median is visualized by the red line. The *P*-value obtained from a paired *t*-test (2-tails): *P*=0.241 shows no significant difference in the prediction power for this out-of-sample cohort.


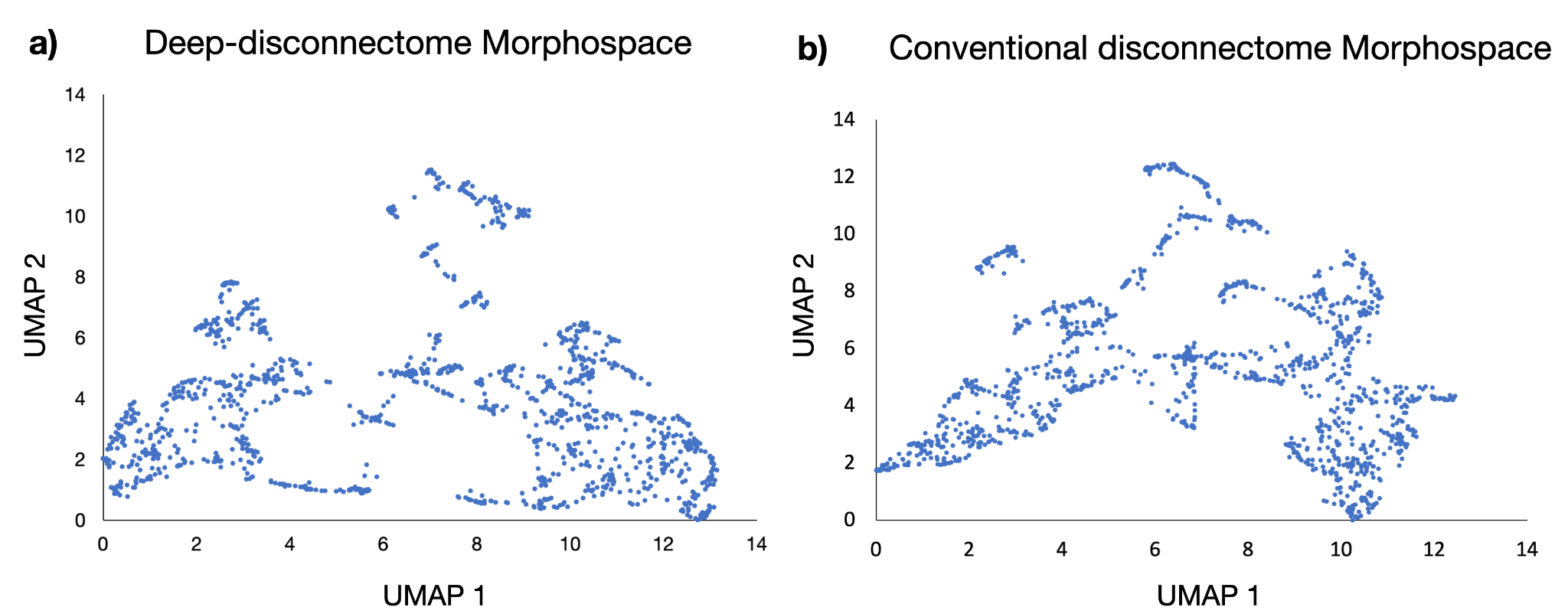


**Supplementary Figure 2:** Comparison of the morphospaces obtained using different disconnectomes. (a) Deep-disconnectome morphospace. (b) Conventional disconnectome morphospace.

**Supplementary References**

1. de Schotten MT, Foulon C, Nachev P. Brain disconnections link structural connectivity with function and behaviour.

2. Foulon C, Cerliani L, Kinkingnehun S, Levy R, Rosso C, Urbanski M, et al. Advanced lesion symptom mapping analyses and implementation as BCBtoolkit. Gigascience. 2018;7(3):giy004.

3. Xu T, Rolf Jäger H, Husain M, Rees G, Nachev P. High-dimensional therapeutic inference in the focally damaged human brain. Brain. 2018 Jan 1;141(1):48–54.
